# Complete Genome Sequences of Methicillin-Resistant Staphylococcus aureus Strains 110900 and 128254, Two Representatives of the CRISPR-Cas-Carrying Sequence Type 630/*spa* Type t4549 Lineage

**DOI:** 10.1128/MRA.00891-20

**Published:** 2020-10-08

**Authors:** Raphael N. Sieber, Søren Overballe-Petersen, Hülya Kaya, Anders R. Larsen, Andreas Petersen

**Affiliations:** a Department of Bacteria, Parasites and Fungi, Statens Serum Institut, Copenhagen, Denmark; University of Southern California

## Abstract

Methicillin-resistant Staphylococcus aureus (MRSA) sequence type 630 (ST630) and *spa* type t4549 is an emerging lineage in Nordic countries, and some representatives carry the CRISPR-Cas system. Here, the complete genome sequences of two isolates from this lineage are presented, comprising chromosomes of 2,918,239 and 2,877,083 nucleotides, respectively, and a 2,473-nucleotide plasmid carrying *erm*(C).

## ANNOUNCEMENT

Methicillin-resistant Staphylococcus aureus (MRSA) of sequence type 630 (ST630) and *spa* type t4549 harbors a staphylococcal cassette chromosome *mec* (SCC*mec*) type V element and a type III-A clustered regularly interspaced short palindromic repeat (CRISPR)-Cas system (1). It has been reported in various Asian countries (2–4) and has recently emerged in Denmark, a low-MRSA-prevalence country, where it was among the most prevalent MRSA lineages in 2018 (5). Therefore, two representative strains of this lineage were sequenced on both a MinION flow cell (Oxford Nanopore Technologies [ONT]) and an Illumina NextSeq 550 sequencer to obtain high-quality reference genome sequences.

Strain 110900 was isolated from a nasal swab from a healthy carrier in the Central Region of Denmark, and strain 128254 was isolated from a skin swab taken from a patient with eczema in the Capital Region of Denmark. Pure cultures of the isolates were submitted to the Danish national reference laboratory as part of the mandatory national surveillance of MRSA. The purity of the isolates was confirmed by streaking onto 5% blood agar plates for visual inspection of the colony morphology prior to *spa* typing as described previously (6) and storage of a single colony of each sample at −80°C. Sample collection and analysis was approved by the Danish Data Protection Agency (protocol number 2001-14-0021).

A 1-ml loop of each frozen single-colony culture was then incubated aerobically overnight at 37°C on 5% blood agar for sequencing. For short-read sequencing, DNA from seven colonies was extracted using a DNeasy blood and tissue kit (Qiagen) and Nextera XT DNA library preparation kit (Illumina, San Diego, USA) for library preparation prior to sequencing with a 2 × 151-bp paired-end midoutput kit. The reads were trimmed with Trimmomatic v0.36 (7) to remove sequencing adaptors and low-quality (Q ≤ 20) read ends. For Nanopore sequencing, DNA from seven colonies was extracted with the GenFind v3 kit (Beckman Coulter) using a DynaMag-2 magnet (Thermo Fisher Scientific). A library was prepared using the rapid barcoding sequencing kit (SQK-RBK004) and sequenced in an R9.4.1 flow cell with a MinION Mk1B instrument (ONT). Fast5 read files were base called and demultiplexed with Guppy v3.2.6 (ONT) in “fast” configuration and quality filtered to a Q value of >10 with NanoFilt v2.5.0 (8) prior to Illumina-Nanopore hybrid or Nanopore-only assembly using Unicycler v0.4.8-beta (9). Default parameters were used for all software unless otherwise specified.

Finally, these assemblies were aligned against each other using BLAST (10) implemented in Geneious Prime v2020.1.2 (Biomatters Ltd., Auckland, New Zealand), and regions of disagreement between the two assemblies were inspected and corrected manually in the locally accurate hybrid assembly. In regions with no hybrid assembly, the Nanopore-only assembly was used. Overlapping ends were identified from the BLAST results, confirmed by read mapping, and trimmed manually prior to closing and rotation of the genome sequences to match the numbering of the Mu50 genome sequence (GenBank accession number BA000017). The closed genomes were error corrected over four iterations of mapping both long and short reads using minimap v2.17 (11) and Geneious mapper (with maximum 3% mismatch, maximum 4 ambiguities, and maximum 10% gaps per match), respectively, followed by single nucleotide polymorphism (SNP) calling using the built-in Geneious algorithm. The final genome and sequencing statistics are presented in Table 1.

**TABLE 1:** Characteristics and accession numbers of the S. aureus ST630/t4549 isolate genomes presented here

Strain	Genome size (bp)	G+C content (%)	No. of genes	Chromosome assembly accession no.	Plasmid assembly accession no.	BioSample accession no.	Sequencing technology	No. of reads	Mean read length (bp)	Sequencing depth (×)	Raw read *N*_50_ (bp)	SRA accession no.^*a*^
110900	2,918,239	32.7	2,942	CP058615	CP058616	SAMN15430096	Illumina	2,757,456	151	160	NA^*b*^	SRR12142664
							MinION	118,776	9,348	201	16,875	SRR12142658–SRR12142661
128254	2,877,083	32.7	2,904	CP058613	CP058614	SAMN15430097	Illumina	2,261,340	151	192	NA	SRR12142665
							MinION	57,843	9,519	111	7,685	SRR12142662–SRR12142663

a MinION read files were split into batches to avoid excessively large file sizes.

b NA, not applicable.

The final genome sequences of strains 110900 and 128254 both contained a plasmid, identified by NCBI BLAST search as identical (0 SNP difference) to a previously published unnamed plasmid of strain UP_1484 (GenBank accession number CP047816) carrying the erythromycin resistance gene *erm*(C). Strain 110900 carried a CRISPR-Cas system associated with the staphylococcal cassette chromosome *mec* (SCC*mec*) type V (5C2&5), while strain 128254 carried an SCC*mec* type V (5C2) and no CRISPR-Cas system.

### Data availability.

The genome sequences of strains 110900 and 128254 were deposited in GenBank under the BioProject accession number PRJNA643825 and the accession numbers presented in Table 1.
